# Effect of melatonin supplementation on sperm quality parameters and expression of antioxidant genes during cold storage of buck semen extenders

**DOI:** 10.14202/vetworld.2024.863-870

**Published:** 2024-04-19

**Authors:** Rini Widyastuti, Sigit Prastowo, Jaswandi Jaswandi, Alkaustariyah Lubis, Rangga Setiawan, Muhammad Rosyid Ridlo, Arief Boediono

**Affiliations:** 1 Department of Animal Production, Faculty of Animal Husbandry, Universitas Padjadjaran, Jl Raya Bandung-Sumedang KM 21, Sumedang, West Java, Indonesia; 2 Department of Animal Science, Faculty of Animal Science, Universitas Sebelas Maret, Surakarta. Indonesia; 3 Department of Reproduction Biotechnology, Faculty of Animal Science, Universitas Andalas. Limau Manis, Pauh, Padang, West Sumatera, Indonesia; 4 Working Group of Genetic Medicine, Faculty of Medicine, Universitas Padjadjaran, Jatinangor, Jl Raya Bandung-Sumedang KM 21, Sumedang, West Java, Indonesia; 5 Department of Bioresources Technology and Veterinary, Vocational College, Universitas Gadjah Mada, Yogyakarta, Indonesia; 6 Department of Anatomy, Physiology and Pharmacology, School of Veterinary Medicine and Biomedical, IPB University, Bogor, West Java, Indonesia

**Keywords:** antioxidant gene expression, melatonin, semen cold storage, sperm quality

## Abstract

**Background and Aim:**

Semen storage is an important reproductive method used in artificial livestock breeding. However, oxidative stress during storage reduces the quality of sperm. Melatonin supplementation in semen storage medium has not been well studied, but it has been shown to protect cells from oxidative stress. Therefore, this study aimed to determine the effect of melatonin supplementation on sperm quality parameters and antioxidant gene expression levels in semen extenders during cold storage.

**Materials and Methods:**

Semen extenders with melatonin concentrations of 0 (control), 0.1, 0.2, and 0.3 mM were added as treatment. The treated semen was then stored at 5°C for 72 h using a cold storage method, and quality parameters, including percentage of progressive motility, membrane integrity, intact acrosome, and DNA integrity, were measured every 24 h. In addition, messenger ribonucleic acid abundance levels of glutathione peroxidase (*GPx*) and superoxide dismutase (*SOD*) genes were sampled after 0 and 72 h of cold storage.

**Results:**

All observed sperm quality parameters decreased with increasing cold storage time; however, 0.2 mM melatonin demonstrated superior protection of sperm quality during cold storage. Gene expression analysis showed that *GPx* levels decreased significantly (p < 0.05) after 72 h in semen without melatonin but not in the melatonin-treated groups. A similar trend was also observed in *SOD*, indicating that exogenous antioxidants effectively protected the sperms.

**Conclusion:**

Melatonin supplementation at 0.2 mM in semen extenders during cold storage maintains sperm quality parameters for up to 72 h because melatonin protects sperm from oxidative stress. These findings can be used to improve the semen storage protocol by combining semen extender and antioxidant.

## Introduction

Sperm preservation or storage is a crucial reproductive technique for human fertility treatments, artificial livestock breeding, and animal conservation [1]. These procedures involve buffer systems and cryoprotective agents [2] and have been widely developed. Oxidative stress, which decreases the viability and fertilizing ability, is the most fundamental challenge [3]. However, oxidation is linked to lipid peroxidation (LPO), DNA fragmentation, and apoptosis, which reduces sperm quality parameters [4]. Minimizing the structural and functional derangement of sperm has been attempted by supplementing the storage medium with antioxidants to reduce the oxidative stress. Antioxidants, such as ascorbic acid, glutathione, coenzyme Q10, melatonin, alpha-tocopherol, and lycopene, have been shown to alleviate reactive oxygen species (ROS)-induced cell damage. By lowering ROS formation, supplementing sperm with antioxidants can increase total motility and viability while decreasing abnormality, as demonstrated by the addition of coenzyme Q10 to buck sperm [5]. In the buffer medium supplemented with alpha-tocopherol and ascorbic acid, LPO levels decreased, whereas glutathione enzymes were enhanced, thereby increasing motility percentage [6]. However, not all antioxidants can be used to counter the adverse effects of ROS in sperm, indicating the need to select the most appropriate antioxidants for preservation.

Melatonin (N-acetyl-5-methoxytryptamine) is a neurohormone synthesized by the pineal gland and is recognized for its potent antioxidant properties. It regulates the circadian rhythm and protects various biological systems against oxidative stress [7]. Melatonin’s free radical scavenging ability can mitigate oxidative damage to spermatozoa during preservation [8]. The previous reports on human [9] and canine [8, 10] sperm cryopreservation showed that melatonin supplementation significantly enhanced frozen sperm parameters, DNA integrity, plasma membrane integrity, intact acrosome, and repressed LPO [10]. Moreover, moderate-level application in fresh and frozen semen improved antioxidant enzymatic activity and reduced mitochondrial oxidative stress, functional capacity, and fertilization [11, 12]. The previous studies by Sun *et al*. [13] and Partyka and Niżański [14] have shown that melatonin supplementation positively affects sperm quality parameters, including motility, vitality, and morphology. It enhances the expression of specific antioxidant genes, thereby influencing the overall antioxidant defense system [15]. However, the integration of melatonin into buffer media as a semen extender during semen storage has not yet been explored, despite its potential implications for animal breeding.

However, information on optimizing melatonin concentration in buck semen storage is limited. Therefore, this study aimed to determine the effect of melatonin on semen extenders, particularly on their ability to scavenge ROS, which could preserve sperm quality parameters. In addition, melatonin supplementation provides an interesting new area of research in reproductive biology. This study will add to the growing body of knowledge by elucidating the potential of this versatile neurohormone, which could transform how we approach semen storage, leading to advances in reproductive treatments, livestock management, and wildlife conservation efforts.

## Materials and Methods

### Ethics approval

The Animal Ethics Committee at Universitas Sebelas Maret in Indonesia approved this study for ethical clearance under record number 1106/EC/11/23. In addition, a veterinarian supervised the study to verify that the animal did not experience pain or suffering during semen sample collection.

### Study period and location

This study was conducted from July to October 2023 at Laboratory Animal Reproduction and Artificial Insemination, Department of Animal Production, Faculty of Animal Husbandry, Universitas Padjadjaran.

### Semen source and extender preparation

Ejaculates (semen) were collected using an artificial vagina, and bucks were teased with an animal teaser. Three bucks (29–30 months old) were used as the semen source. All bucks are reared in the same barn, fed the same diet, and have access to water *ad libitum*. Ejaculates were observed for their volume and wave motion scores after collection. Wave rates at ≥3 (scores 0–5) were then selected and sample preparation was then conducted. The base medium of the semen extender was egg yolk Tris buffer (3.63 g Tris, 1.99 g citric acid, 0.50 g fructose, 14% v/v egg yolk, 6% glycerol v/v, 100.000 IU penicillin, and 100 mg streptomycin). All samples were diluted to a total volume of 100 mL in distillate water. The final melatonin concentrations were set at 0 (control), 0.1, 0.2, and 0.3 mM, and the pH of the semen extender was adjusted to 7.2–7.4. The semen and extender were gently mixed at a final sperm concentration of 500 × 10^6^ sperm/mL once the media were ready. Semen cold storage was performed at 5°C for 72 h, and sperm quality parameters were evaluated every 24 h.

### Sperm quality evaluation

#### Motility parameter

Sperm motility in all samples was assessed subjectively using five microscopic fields. A drop of diluted semen was placed on a pre-warmed glass slide (37°C), which was then covered with a pre-warmed coverslip. Progressive motility was then subjectively scored from 0% to 100%.

### Sperm membrane integrity

Sperm plasma membrane integrity was assessed using hypo-osmotic swelling (HOS) test. The HOS solution was prepared in 100 mL of distilled water with 0.735 g of sodium citrate and 1.351 g of fructose. Approximately 50 μL of semen was added to 500 μL of HOS solution, which was incubated at 37°C for 30 min [16]. After incubation, 5 μL of the incubated semen was then stained with 2% eosin on a glass slide and air-dried before examination under a 400× magnification microscope. A total of 200 sperms with clear heads and swollen tails were observed, indicating a biochemically active sperm membrane. On the other hand, pink heads and unswollen tails are signs of damaged and inactive sperm membrane [16]. Active versus inactive sperm membranes were calculated based on the total sperm count as a percentage (%).

### Sperm intact acrosome

A staining solution containing 2% (w/v) fast green FCF and 0.8% (w/v) eosin B dissolved in a glutamate-based extender diluent [16] at pH = 7.35 was used to determine sperm acrosome status. In total, 20 μL of the diluted semen was smeared with 10 μL of the staining solution and air-dried at room temperature (25°C). Examination was then performed using an Olympus CX21 microscope (Tokyo, Japan). On the basis of the eosinophilia level, a differential count was performed on 200 stained or unstained sperm [16]. The number of stained or unstained sperm was converted into percentages.

### Sperm DNA integrity

Acridine orange (AO) assay was used to determine the DNA integrity of the sperm. Semen was smeared on glass slides, air-dried, and fixed overnight in freshly prepared Carnoy’s solution containing a 3:1 mixture of methanol and glacial acetic acid. The samples were air-dried for 5 min before staining with an AO solution consisting of 10 mL of 1% AO in distilled water, 40 mL of 0.1 M citric acid, and 2.5 mL of 0.3 M Na_2_HPO_4_.2H_2_O. The slides were carefully washed with distilled water and kept in a cold, dark place for further evaluation [17]. The percentage of sperms with normal DNA was calculated by counting at least 200 sperms using a fluorescent microscope (BX41; Olympus, Tokyo, Japan) at 400× magnification and fluorescence excitation at 450–490 nm. Normal sperm with intact double-stranded DNA were dyed green, whereas those with denatured DNA fluoresced red or orange.

### Gene expression analysis

#### Sperm RNA extraction

Three biological replications of semen samples from 0 and 72 h of each melatonin treatment were extracted for their sperm RNA using the RNeasy mini kit (Qiagen, USA) according to the protocol with minor modifications. Modification was performed during lysis using a 20-g needle syringe. After extraction, RNA concentration and purity were determined using a NanoDrop ND-2000C (Thermo Fisher Scientific, MA, USA).

### cDNA synthesis and real-time polymerase chain reaction (RT-PCR)

cDNA reverse transcription of 200 ng of total RNA was performed for each sample using ReverTra Acetum qPCR RT Master Mix with gDNA Remover (Toyobo, Osaka, Japan) according to the manufacturer’s instructions. To determine the expression of each intended gene, 2 μL of cDNA was added to the master mix, containing 5 μL of THUNDERBIRD SYBR qPCR Master Mix (Toyobo), 3 μL of nuclease-free water, and 12.5 pmol forward and reverse primers. Subsequently, quantitative RT-PCR was performed using the StepOne Real-Time PCR System (Thermo Fischer Scientific), and predenaturation at 95°C for 1 min, 40 denaturation cycles for 15 s, and annealing and extension at 55°C for 1 min were performed. The glutathione peroxidase (*GPx*) and superoxide dismutase (*SOD*) messenger ribonucleic acid (mRNA) abundance levels were quantified using their own-designed primers. Primer design was performed using Primer 3.0 (https://primer3.ut.ee/), and the reference sequences were based on the National Center for Biotechnology Information (www.ncbi.nlm.nih.gov) database. Table-1 shows the set of primers per gene. Actin beta was selected as the housekeeping gene, and the expression of each gene was calculated using the 2^−ΔΔCT^ method.

**Table-1:** Primers used for quantitative reverse-transcription polymerase chain reaction of genes in sperm.

Gene	Primer sequences (5’-3’)	Annealing temperature (°C)	Product size (bp)	Accession number
GPx				
Forward	GTCTCCTGGAACTTCGAGA	55	216	XM_004018462.5
Reverse	CGTAGGTTTAGAGGGAACAC			
SOD				
Forward	GGCAGAGGTGGAAATGAAG	55	115	NM_001145185.2
Reverse	ACTAGGACTCAGACCATGTC			
ACTB				
Forward	GACTGTTAGCTGCGTTACAC	55	206	AY141970.1
Reverse	CCCAAAGTTCTACAGTGTGG			

GPx=Glutathione peroxidase, SOD=Superoxide dismutase, ACTB=Actin beta

### Statistical analysis

In this study, all sperm quality parameters are presented as mean ± standard deviation. The experiment was designed as factorial with two factors, namely, melatonin concentration (mM) and cold storage time (h). Analysis of variance was employed for statistical comparison between factors, and any significant differences were then evaluated with *post hoc* analysis using Duncan’s multiple range test. Gene expression data analyses of *GPx* and *SOD* with respect to mRNA abundance were performed before (0 h) and after (72 h) cold storage using the t-test. All data analysis and graph presentation were performed with the help of R-4.3.1 free software environment for statistical computing. The statistical difference was set at p < 0.05.

## Results

**Figure-1:**
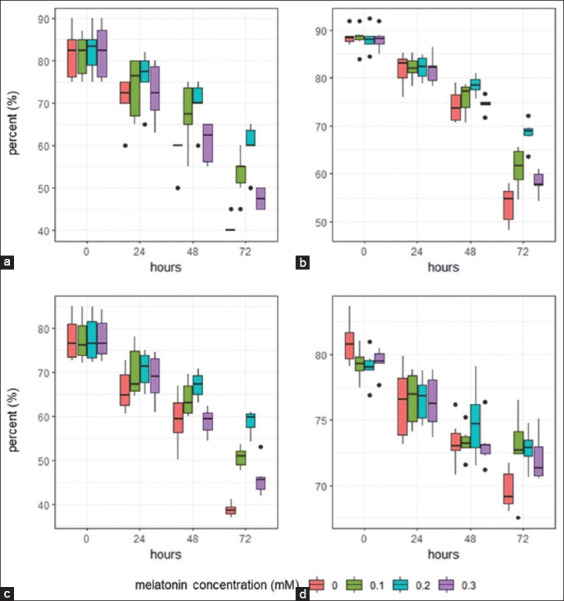
Data distribution of the sperm quality parameters during cold storage in different melatonin concentrations. (a) Motility, (b) membrane integrity, (c) intact acrosome, and (d) DNA integrity.

As shown in Table-2, melatonin concentration and cold storage time significantly affected sperm parameters, including motility, membrane integrity, and percentage of intact acrosomes (p < 0.05) but not DNA integrity. Therefore, the sperm quality parameter in this study was either influenced or determined by both factors. Melatonin factor concentrations affected sperm quality in all observed parameters, except DNA integrity. The 0.2 mM melatonin group showed better (p < 0.05) protection over 72 h of cold storage than the control and other groups (0.1 and 0.3 mM). This is illustrated in the distribution of sperm quality data in Figures-1a–d (green box plot).

**Table-2:** Effect of melatonin concentration and cold storage time on sperm quality parameters.

Factors		Motility	Membrane integrity	Acrosome intact	DNA integrity

Effect of interaction					

Melatonin concentration (mM)	Cold storage time (h)				
0	0	81.67 ± 6.06^a^	88.70 ± 1.74^a^	77.56 ± 5.03^a^	80.92 ± 1.68^a^
0.1	0	81.33 ± 5.09^a^	88.26 ± 2.55^a^	77.34 ± 4.95^a^	79.26 ± 1.20^a^
0.2	0	82.50 ± 5.39^a^	88.11 ± 2.61^a^	77.54 ± 5.25^a^	79.06 ± 1.34^a^
0.3	0	82.17 ± 6.49^a^	88.18 ± 2.27^a^	77.65 ± 4.75^a^	79.42 ± 0.98^a^
0	24	70.83 ± 5.85^bc^	81.80 ± 3.51^b^	65.94 ± 4.84^bc^	76.30 ± 2.76^b^
0.1	24	73.83 ± 7.36^bc^	82.04 ± 2.39^b^	70.01 ± 6.00^b^	76.65 ± 2.05^b^
0.2	24	76.17 ± 6.18^ab^	82.14 ± 2.45^b^	70.61 ± 4.08^b^	76.58 ± 1.70^b^
0.3	24	72.67 ± 6.86^bc^	81.74 ± 2.98^b^	68.75 ± 5.39^bc^	76.35 ± 2.11^b^
0	48	58.33 ± 4.08^de^	74.18 ± 3.41^d^	59.30 ± 5.93^d^	73.32 ± 1.80^cd^
0.1	48	67.50 ± 7.58^c^	75.83 ± 3.21^cd^	63.97 ± 3.96^cd^	73.31 ± 1.19^cd^
0.2	48	70.00 ± 5.48^bc^	78.50 ± 1.94^bc^	67.05 ± 3.06^bc^	74.83 ± 2.78^bc^
0.3	48	60.83 ± 4.92^d^	74.47 ± 1.61^b^	58.84 ± 2.96^d^	73.18 ± 1.72^cd^
0	72	40.83 ± 2.04^g^	53.60 ± 3.98^g^	38.85 ± 1.53^f^	69.66 ± 1.52^e^
0.1	72	53.33 ± 5.16^ef^	61.16 ± 4.31^f^	50.70 ± 2.21^e^	72.71 ± 2.99^cd^
0.2	72	60.00 ± 5.48^de^	68.48 ± 2.80^e^	58.77 ± 2.64^d^	72.81 ± 1.42^cd^
0.3	72	47.50 ± 2.74^f^	58.10 ± 2.43^f^	45.92 ± 3.96^e^	72.06 ± 1.83^d^
p-value		0.02	6.99×10^−7^	1.27×10^−4^	0.10
Effect of melatonin concentration (mM)					
0		62.92 ± 16.15^c^	74.57 ± 13.78^c^	60.41 ± 15.00^c^	75.05 ± 4.61
0.1		69.00 ± 12.09^ab^	76.82 ± 10.70^b^	65.51 ± 10.83^b^	75.48 ± 3.29
0.2		72.17 ± 9.98^a^	79.31 ± 7.64^a^	68.49 ± 7.80^a^	75.82 ± 2.94
0.3		65.79 ± 14.22^bc^	75.62 ± 11.67^bc^	62.79 ± 12.72^c^	75.25 ± 3.34
p-value		1.21×10^−6^	1.21×10^−6^	5.69×10^−8^	0.54
Effect of cold storage time (h)					
0		81.92 ± 5.41^a^	88.31 ± 2.18^a^	77.53 ± 4.66^a^	79.67 ± 1.45^a^
24		73.38 ± 6.45^b^	81.93 ± 2.68^b^	68.83 ± 5.12^b^	76.47 ± 2.05^b^
48		64.17 ± 7.17^c^	75.74 ± 3.03^c^	62.29 ± 5.20^c^	73.66 ± 1.95^c^
72		50.42 ± 8.20^d^	60.33 ± 6.41^d^	48.56 ± 7.83^d^	71.81 ± 2.30^d^
p-value		2.00×10^−16^	2.00×10^−16^	2.00×10^−16^	2.00×10^−16^

All sperm quality parameters in the table are shown as mean ± SD. ^a,b^ Values followed with different superscripts in the same column showed significant difference (p < 0.05)

**Figure-2:**
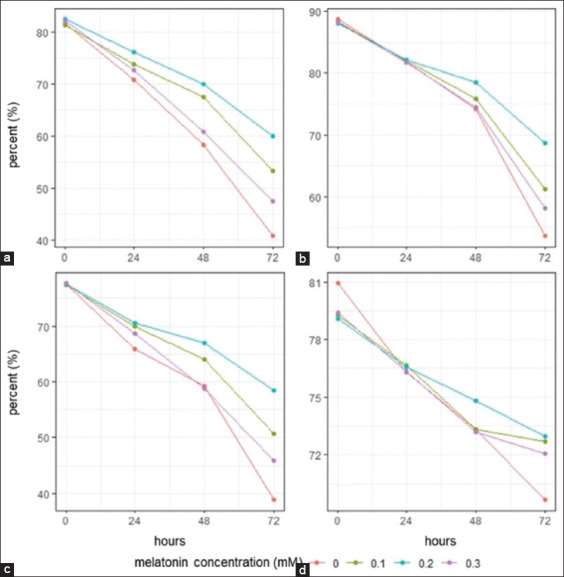
Sperm quality parameter decline during cold storage in different melatonin concentrations. (a) Motility, (b) membrane integrity, (c) intact acrosome, and (d) DNA integrity.

With regard to the effect of cold storage time, sperm quality parameters significantly (p < 0.05) declined from 0 to 72 h in all melatonin groups (Table-2 and Figures-2a–d). A decrease in sperm quality over the observation period compared with the starting point (0 h) was observed with cold storage time. The melatonin groups showed higher motility (p < 0.05) compared with the control group (Figure-2a). Cold storage of semen using 0.2 mM melatonin for up to 72 h was found to be the best dosage to preserve motility (60%). A similar trend was observed for membrane integrity, intact acrosome, and DNA integrity percentage (Figures-2b–d).

Table-2 shows the percentage of HOS test-reactive sperm, which means intact membrane during cold storage. Melatonin supplementation with semen extender did not affect the sperm membrane in the first 24 h. However, melatonin treatment reduced membrane damage at 48–72 h compared with the control (Figure-2b). Furthermore, compared with the 0.1 and 0.3 mM melatonin groups, the 0.2 mM melatonin group had a higher (p < 0.05) percentage of intact sperm membrane during cold storage.

Data on intact acrosomes showed that cold storage generally induced loose acrosomes in the control and melatonin-treated groups throughout the study period. The melatonin group had higher (p < 0.05) acrosomal integrity (Table-2) than the control group during the cold storage period of 24–72 h. As shown in Figures-1c and 2c, the 0.2 mM melatonin group showed a higher number of intact acrosomes than the other groups.

DNA integrity was reduced in all groups during cold storage (Table-2 and Figure-2d). However, melatonin at all concentrations was associated with superior DNA integrity in the sperm. These results demonstrate that the treated sperms were protected from cooling at all observation times compared with the controls.

**Figure-3:**
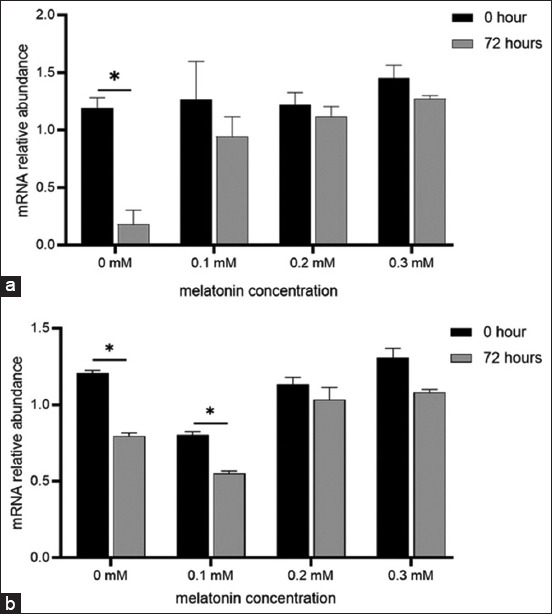
Gene expression of *GPx* (a) and *SOD* (b) in sperm before (0 h) and after (72 h) cold storage at different melatonin concentrations (*p < 0.05). *GPx*=Glutathione peroxidase, *SOD*=Superoxide dismutase.

To determine the effect of melatonin on gene expression, the mRNA abundance level analysis of *GPx* and *SOD* was performed in sperm samples at 0 h (before) and 72 h (after) of storage *GPx* protects cells against ROS-induced damage, whereas SOD scavenges ROS through antioxidant mechanisms. As shown in Figure-3a, GPx mRNA abundance was significantly reduced (p < 0.05) after 72 h in the control group (0 mM). However, in the melatonin groups (0.1, 0.2, and 0.3 mM), expression was maintained after 72 h of storage. Figure-3b shows the significant reduction (p < 0.05) of the SOD mRNA abundance level in the 0 and 0.1 mM melatonin groups after 72 h.

After 72 h, the sperm gene expression levels of *GPx* and *SOD* were not significantly different between the 0.2 and 0.3 mM melatonin groups (p > 0.05). These results show that the level of endogenous antioxidants can be enhanced to combat ROS from exogenous sources. As demonstrated by the *GPx* and *SOD* expression, semen extenders supplemented with melatonin maintained all sperm quality parameters (Table-2). However, the 0.2 mM melatonin group showed the best protection, as depicted in Figures-1 and 2.

## Discussion

Sperm preservation is an effective technique for maintaining genetic diversity and conserving endangered species. In the field of animal husbandry, this technique plays a key role in the production of genetically superior breeding animals and contributes to the development of artificial reproduction technology. A previous study by Pintus and Ros-Santaella [18] has shown that ROS are generated and accumulated during sperm preservation in rams, humans, bulls, and rabbits. Sperms are vulnerable to ROS stress due to the high unsaturated fatty acid content in their membrane [19]. This accumulation can induce LPO in the sperm membranes, resulting in damage and loss of motility [20]. Therefore, the addition of antioxidants to semen extenders is recommended to minimize the negative effect of ROS and maintain sperm quality during preservation.

On the basis of these results, sperm quality parameters decreased with cold storage time (Figure-2). For up to 72 h, supplementation with 0.2 mM melatonin in semen extenders inhibited the decrease in progressive motility, membrane integrity, and intact acrosome. This is consistent with the results of a previous study by Palhares *et al*. [21] that showed that melatonin protects against oxidative stress and consequently maintains sperm motility. Melatonin has a potential cryoprotective effect on mitochondrial functioning, which is essential for generating energy from intracellular adenosine triphosphate reserves, thereby enhancing sperm motility [22]. Melatonin supplementation effectively sustains sperm motility by maintaining the integrity of the acrosome and plasma lemma [23].

Sperm and acrosome membranes protect the cytosolic contents and physiology. Excessive ROS accumulation damages membrane integrity during preservation, resulting in disturbance of protein structures, lipids, and nucleic acids. This triggers peroxidation chain reactions, causing damage to membrane components, particularly lipids [24]. In addition, excessive ROS potentially induces hyperactivation due to loose acrosomes, reducing the sperm-fertilizing capacity during *in vivo* fertilization or conventional *in vitro* fertilization. The acrosome is critical for binding and fusing with the plasma membrane of the oocyte [25]; hence, any disruption will alter the sperm’s ability to fertilize the oocyte.

Melatonin supplementation in semen extenders prevented a drastic reduction in sperm membrane integrity and intact acrosome for up to 72 h (Figure-3). Melatonin facilitates transport across the plasma membrane and uniform distribution in the cytosol to reduce LPO and maintain structural integrity of sperm [23]. In addition, melatonin accumulates in the mitochondria to maintain their function and activity [26]. It binds directly to specific melatonin receptors, mainly melatonin receptor 1 and melatonin receptor 2 in the sperm plasma membrane, interacting and regulating endogenous antioxidants, and resulting in enhanced sperm function and regulation of apoptotic-like alterations [27].

Moreover, melatonin supplementation preserved DNA integrity in semen extenders (Table-2 and Figure-2). Free radicals generated by oxidative stress may adversely affect sperm DNA during preservation. Melatonin and its metabolites scavenge free radicals and protect sperm from oxidative DNA modification [28, 29]. It increases resistance to fragmentation, prevents degradation, and enhances viability and functions [30]. Melatonin also protects against DNA damage and improves sperm quality by altering the expression of antioxidant genes [31]. This was demonstrated by *GPx* and *SOD* expression profiling (Figure-3 in this study).

Antioxidant enzymes play a crucial role in cell defense by maintaining an appropriate ROS level. A previous study by Pool *et al*. [12] showed that sperm intermembrane can generate excessive singlet oxygen radical O_2_•− and hydrogen peroxide (H_2_O_2_) during storage. *SOD* converts singlet oxygen radicals into H_2_O_2_ and molecular oxygen (O_2_), and GPx converts H_2_O_2_ into water and lipid alcohols [32]. In this study, *GPx* mRNA levels were insignificantly reduced (Figure-3a) in melatonin-treated sperm after 72 h of cold storage, whereas *SOD* mRNA abundance (Figure-3b) was maintained or enhanced by 0.2 and 0.3 mM melatonin, respectively. These results suggest that melatonin effectively protects the sperm plasma membrane against LPO and protects DNA from oxidative damage, thus preventing the reduction of *GPx* mRNA levels.

The significantly decreased mRNA SOD levels (Figure-3b) at 0 and 0.1 mM could be attributed to the leakage of intracellular enzymes caused by membrane damage. This may have been influenced by an increase in LPO, thereby decreasing the sperm quality [33]. Moreover, decreased mRNA expression of antioxidant genes such as *GPx* and *SOD* might result in apoptosis [34], as shown by the higher number of sperm with membrane damage, acrosome loss, and broken DNA (Table-2).

Melatonin supplementation in semen extenders can directly affect the quality and function of sperm through protection against injury. This study showed that the right melatonin concentration is crucial for obtaining the desired effect. A 0.2 mM concentration was identified as the best dosage to protect sperm during cold storage (Figures-1 and 2). However, a further increase to 0.3 mM did not increase the antioxidant effect due to the toxic effects of higher concentrations. Melatonin protects sperm from oxidative stress-induced damage during cold storage of semen through several mechanisms, including direct scavenging of excessive ROS, membrane integrity stabilization, improvement of endogenous antioxidant defenses, apoptosis inhibition, and DNA preservation. In addition, treatment (melatonin supplementation) maintained *GPx* and *SOD* mRNA expression during storage. Overall, this study demonstrates that melatonin protects sperm from damage during cold storage by enhancing antioxidant defense mechanisms.

## Conclusion

The addition of melatonin to semen extenders during cold storage has a significant effect on sperm quality parameters for up to 72 h. Notably, administration of 0.2 mM melatonin during cold storage of semen proved to be the most effective way to preserve sperm quality by maintaining endogenous antioxidant levels. This treatment regimen significantly protected sperm motility, membrane integrity, and acrosome integrity while maintaining *GPx* and *SOD* gene expression levels. These findings highlight the potential of adding melatonin to semen extenders to improve sperm quality and lifespan during storage.

## Authors’ Contributions

RW and SP: Designed the study, carried out the experiment, data interpretation and drafted the original manuscript. AL, MRR, RS, JJ, and AB: Data curation and performed in-depth review of work to assess its significant intellectual content. All authors have read, reviewed, and approved the final manuscript.
